# Advances in Non-Small Cell Lung Cancer (NSCLC) Treatment—A Paradigm Shift in Oncology

**DOI:** 10.3390/ph17020246

**Published:** 2024-02-13

**Authors:** Azhar Ali

**Affiliations:** Cancer Science Institute of Singapore, MD6, Center for Translational Medicine, #12-01, National University of Singapore, 14 Medical Drive, Singapore 117599, Singapore; csiazhar@nus.edu.sg

Non-Small Cell Lung Cancer (NSCLC) management remains a formidable challenge in the field of oncology, representing a significant global health burden. However, recent years have witnessed a revolutionary transformation in the landscape of NSCLC treatment. Breakthroughs in understanding the molecular intricacies of this disease, coupled with innovative therapeutic approaches, have ushered in a new era of hope for patients and healthcare providers alike. The advent of targeted therapies has marked a pivotal turning point in NSCLC management. Traditional chemotherapy, with its broad-spectrum approach, often inflicts collateral damage on healthy cells, causing unfavourable side effects. However, subsequent identification of specific genetic mutations driving NSCLC has paved the way for more precise and effective treatments. One of the ground-breaking discoveries in NSCLC is the role of activating epidermal growth factor receptor (EGFR) mutations in tumour progression [1]. EGFR inhibitors have demonstrated remarkable efficacy in tumours harbouring EGFR mutations by selectively targeting cancer cells with mutated EGFRs, thus minimising harm to normal cells. Other targeted therapies which have been developed against the diverse genomic landscape in NSCLC include anaplastic lymphoma kinase (ALK) inhibitors for gene rearrangements [2] and mutations in MET [3], ROS1 [4], BRAF [5] and KRAS [6], which have led to the development of targeted inhibitors, including crizotinib, alectinib, entrectinib, dabrafenib and sotosarib, respectively, providing tailored options for patients with these specific genetic alterations. Immunotherapy is a revolutionary approach in NSCLC treatment, capitalising on the body’s immune system to recognise and eliminate cancer cells. Programmed death-1 (PD-1) and programmed death-ligand 1 (PD-L1) are key players in immune evasion by cancer cells. Immune checkpoint inhibitors (ICIs), such as Pembrolizumab, Nivolumab and Atezolizumab, have demonstrated unprecedented success in extending survival and improving the quality of life of NSCLC patients [7]. Inhibiting these checkpoints unleashes the immune system’s ability to recognise and attack cancer cells. Pembrolizumab, a PD-1 inhibitor, has shown remarkable results, particularly in NSCLC patients with high PD-L1 expression [8]. 

Despite the unprecedented progress in NSCLC treatment, challenges persist. Resistance mechanisms to targeted therapies and immunotherapy, the identification of novel therapeutic targets and oncogenic pathways, and the development of strategies for overcoming tumour heterogeneity are ongoing areas of research. New emerging targets for patients with no known driver mutations have been identified for targeted therapy in NSCLC. These new targets include gene arrangements in neuregulins (NRGs) [9] and CAP-Gly domain-containing linker protein 1- leukocyte receptor tyrosine kinase (CLIP-LTK) [10] and mutations in discoidin domain receptors (DDRs) [11], phosphatase and tensin homolog (PTEN) [12], nuclear factor erythroid-2-related factor-2/kelch-like ECH-associated protein 1 (NFE2L2/KEAP1) [13] and serine threonine 11/liver kinase B1 (STK11/LKB1) [14] to facilitate the development of effective therapies for advanced NSCLC lacking known oncogenic drivers. Combinations of checkpoint inhibitors, along with chemotherapy or other targeted agents, are being investigated to enhance the effectiveness of immunotherapy and broaden its applicability across different patient populations. Currently, there is a lack of solid biomarkers to predict sensitivity to immunotherapy. In this Special Issue, the combination therapy of Pembrolizumab and chemotherapy showed significant benefit in progression-free survival (PFS) in treatment-naïve advanced NSCLC patients with high PD-L1 expression and a low neutrophil-to-lymphocyte ratio when compared with monotherapy [15]. It is important to note that the majority of patients with high tumour PD-L1 do not respond to ICIs; hence, the identification of a low neutrophil-to-lymphocyte ratio is crucial in patient selection for a more effective ICI response. Nevertheless, the landscape of NSCLC immunotherapy is continually evolving, with ongoing research to deconvolute mechanisms of resistance and exploring combination strategies to improve its efficacy. Real-world studies are essential for understanding how immunotherapy drugs work in diverse patient populations by providing insights into the effectiveness, safety and tolerability of immunotherapies outside the controlled environment of clinical trials. In this Special Issue, the treatment efficacy of Nivolumab (anti-PD-1 antibody) and Atezolizumab (anti-PD-L1 antibody) was evaluated by Alonso-Garcia et al. [16] in a cohort of 158 previously treated patients with stage IV or recurrent NSCLC. In this retrospective study, the authors reported that there were no significant differences in PFS and overall survival (OS) outcomes between Nivolumab and Atezolizumab and concluded a more favourable toxicity profile for Atezolizumab from safety profiles. In a similar study, data collected from 1607 patients with advanced NSCLC treated with second-line treatment with Nivolumab, Pembrolizumab or Atezolizumab were used to compare the effectiveness and cost-effectiveness of these ICIs [17]. In this study, no significant difference in OS was observed between these groups, and Atezolizumab was associated with the most favourable cost-effectiveness profile. These data can provide invaluable information for future clinical decision-making, improving treatment guidelines and enhancing patient care.

The development of new technologies and alternative paradigms has contributed significantly to improving the landscape of NSCLC treatment. Traditional tissue biopsies have limitations, especially in obtaining real-time information on tumour dynamics. Liquid biopsies, which analyse circulating tumour DNA (ctDNA) in the bloodstream, are now available as a non-invasive and dynamic approach to monitor disease progression, identify resistance mutations, and help guide treatment decisions [18]. Chimeric Antigen Receptor T cell (CAR-T) therapy, in the early stages of exploration for NSCLC, holds promise. Through the genetic modification of a patient’s own T cells to express a receptor targeting specific cancer antigens, CAR-T therapy has demonstrated ground-breaking success in haematological malignancies and is now being investigated for its potential in solid tumours [19]. Novel approaches such as inhalation therapy [20], improving drug solubility for delivery [21] and drug repurposing [20,22] are discussed in Review articles in this Special Issue. The promise of drug repurposing and combination therapy was demonstrated by Barrious-Bernal et al. [23], in which Metformin, an anti-diabetic drug, when combined with EGFR TKI-Afatinib, demonstrated additive to synergic anti-tumour effects in TKI-resistant lung cancer cells. Alternative paradigms proposed for NSCLC therapy in this Special Issue include targeting nuclear receptors [24], DNA damage responses [25] and inflammation [20]. Inflammation is often associated with cancers, including NSCLC, where it promotes malignant transformation and tumour initiation. In this Special Issue, Sorafenib, an FDA-approved multi-kinase inhibitor, was shown to be capable of reducing diethylnitrosamine (DEN)-induced lung pre-cancer lesions through the downregulation of inflammation-associated JNK and COX-2 signalling as well as TNF and IL-1β protein levels in rat models [26]. Another challenge in NSCLC treatment is the lack of reliable prognostic markers, which has severely impeded effective treatment planning and management of NSCLC patients. In this Special Issue, pro-inflammatory chemokine CXCL12 expression was evaluated to determine its value as a prognostic marker for tumour recurrence in a cohort of 82 NSCLC patients [27]. The authors observed that low CXCL12 expression was associated with significant prolonged PFS and OS, while, in patients with high tumour CXCL12 expression, PFS and OS were significantly improved after adjuvant chemotherapy compared with those in untreated patients. These findings are crucial for ongoing efforts to identify a reliable prognostic marker for NSCLC.

In conclusion, current advances in NSCLC treatment represent a triumph of scientific ingenuity and a beacon of hope for patients and their families. Targeted therapies, immunotherapy and emerging technologies have collectively ushered in a new era of precision medicine, transforming NSCLC from a once ominous diagnosis to a condition with increasingly optimistic prognoses. Additionally, access to these advanced treatments, cost considerations and potential long-term side effects warrant attention as we navigate the evolving landscape of NSCLC management. This Special Issue serves as a platform for researchers to present their work as we continue to unravel the complexities of NSCLC biology. The collaborative efforts of researchers, clinicians and pharmaceutical innovators can shape the future of NSCLC treatment and bring us closer to a day when this formidable adversary can be conquered. 
